# Rib Osteomyelitis in a Pediatric Patient: Case Report and Review of the Literature

**DOI:** 10.5811/cpcem.2018.9.39481

**Published:** 2018-10-16

**Authors:** Philip D. Nibley, Chadd K. Kraus

**Affiliations:** * Vituity Emergency Medicine, Sacramento, California; † Geisinger Health, Department of Emergency Medicine, Danville, Pennsylvania

## Abstract

We present a case report and review of the literature of rib osteomyelitis in a pediatric patient presenting to the emergency department (ED) with fever and increased work of breathing. The patient was seen on a return visit to the ED after discharge with presumed viral illness approximately 12 hours prior. On the second ED visit, there was concern for occult bacteremia, and work-up ultimately revealed a subperiosteal abscess with rib osteomyelitis, a rare etiology for fever in the pediatric patient. The patient was treated with antibiotics, had surgical debridement, and fully recovered.

## INTRODUCTION

Fever is a common presenting complaint of pediatric patients in the emergency department (ED). Fever can represent non-specific viral syndromes or serious bacterial infections. Occult bacterial infections, such as osteomyelitis, are less common than viral infections but can frequently elude diagnosis. We present a case report and review of the literature of rib osteomyelitis in a pediatric patient presenting to the ED with fever and increased work of breathing.

## CASE REPORT

A nine-month-old male presented to the ED with a four-day history of fever and increased work of breathing. He was first seen by his primary care physician with the onset of symptoms. His mother later took him to the ED where he had a fever but no respiratory distress. He was treated symptomatically and discharged with presumptive diagnosis of viral illness. He returned to the ED approximately 12 hours after discharge with abdominal pain and recurrence of his fever. His parents noted that the patient seemed to be in pain and had grunting with expiration. He also had decreased oral intake, but was still making wet diapers.

His parents also reported cyclical periods of crying during which he seemed uncomfortable. They noted that he seemed to be pale with decreased energy and activity from baseline. Mom also reported no bowel movements for the prior 24 hours, with the patient normally having 2–3 bowel movements daily. Parents denied any cough, congestion, wheezing, stridor, vomiting, or rash. He had no past medical history, and his vaccinations were up to date.

On exam the patient had a rectal temperature of 101.5 degrees Fahrenheit, heart rate of 187 beats per minute, respiratory rate of 36 breaths per minute, and oxygen saturation of 99% on room air. He appeared to be developmentally appropriate and in moderate distress with pale skin; he exhibited no cyanosis, rash, or lesions. He had an expiratory grunt with each breath. No cardiac murmur was appreciated and the lungs were clear without wheezes. The abdomen revealed no focal tenderness. Muscle tone was within normal limits. His neurological exam was without focal deficits and age appropriate.

Labs showed a white blood cell count of 17.0 K/microliter (mcL) (normal 6.0–17.5 K/mcL), C-reactive protein of 13.16 milligrams per deciliter (mg/dL) (normal 0–0.80 mg/dL), lactic acid of 1.8 millimoles/L. Urinalysis was unremarkable. The chest radiograph and ultrasound of the abdomen were unremarkable. An electrocardiogram showed sinus tachycardia. Blood cultures were ordered and the patient was started on empiric antibiotics (piperacillin/tazobactam) in discussion with the pediatric hospitalist service. He was admitted for additional evaluation of fever of unknown origin. Blood cultures were initially positive for methicillin-susceptible *Staphylococcus aureus* (MSSA). An echocardiogram ordered for suspicion of endocarditis was normal. Antibiotics were continued, and the patient improved clinically; however, his parents noted during his hospitalization that he seemed to be in pain when he was picked up, particularly in his axillae.

**Image 1:**
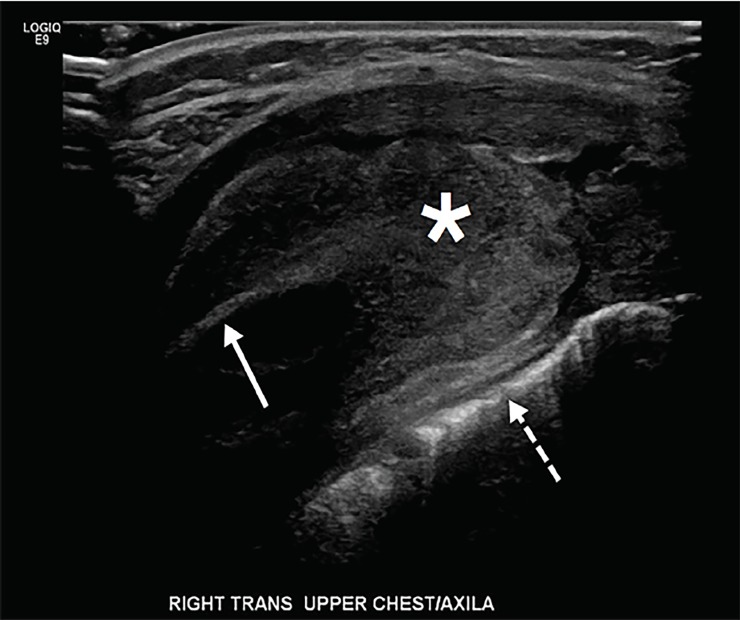
Formal ultrasound of right upper chest/axilla showing rib (arrow), lung border (dashed arrow), and periosteal abscess (*).

**Image 2:**
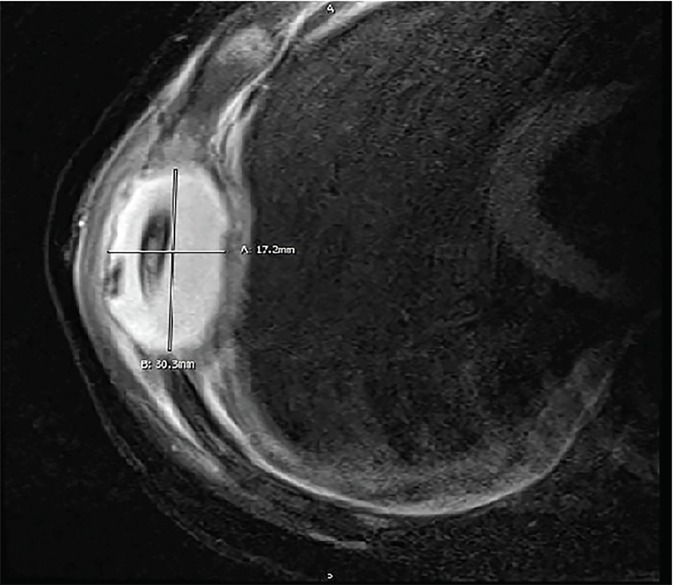
Magnetic resonance imaging (axial view: T1, T2 fat saturation technique) showing 17.2 × 30.3 mm periosteal abscess of rib.

Repeat blood cultures obtained after antibiotic therapy were negative, and the patient’s fevers were less frequent. He was transitioned to oral cephalexin and observed. On hospital day six, the patient’s mother noted a 2×4 centimeter tender, non-erythematous mass in the right axilla. Formal ultrasound showed a soft tissue mass adjacent to the rib, without a definitive fluid collection (Image 1). The patient was transferred to a specialized pediatric hospital where magnetic resonance imaging (MRI) revealed that the axillary mass was consistent with osteomyelitis and subperiosteal abscess of the lateral seventh rib (Image 2). The patient had operative debridement and thereafter made a full recovery with discharge home on hospital day 12.

## DISCUSSION

Rib osteomyelitis in pediatric patients is rare, with less than 60 cases reported in the literature.^1^,^3^ It has been described in association with tuberculosis,^4^ via hematogenous spread,^5^ and as a sequela of child abuse.^6^ Our case illustrates that uncommon and rare causes of fever of unknown origin, such as osteomyelitis, should be considered in the evaluation of pediatric patients presenting to the ED. The etiology of osteomyelitis in this case was not clear, and could have been the result of hematogenous spread, although no primary site of infection was evident. Most cases of rib osteomyelitis are *S. aureus*,^2^,^7^,^8^ although other organisms including *Streptococcus pneumonia* and Group A beta hemolytic *Streptococcus* have also been implicated.^9^–^11^

**CPC-EM Capsule**

What do we already know about this clinical entity?

In pediatric patients presenting to the emergency department (ED) with fever, occult bacterial causes, such as osteomyelitis, are less common than viral causes but frequently elude diagnosis.

What makes this presentation of disease reportable?

Rib osteomyelitis in pediatric patients is rare, with less than 60 cases reported in the literature.

What is the major learning point?

The diagnosis of rib osteomyelitis requires a high index of suspicion by the emergency physician, especially because the presentation can be non-specific.

How might this improve emergency medicine practice?

Uncommon and rare causes of fever of unknown origin, such as osteomyelitis, should be considered in the evaluation of pediatric patients presenting to the ED.

## CONCLUSION

The diagnosis of rib osteomyelitis requires a high index of suspicion by the emergency physician, especially because the presentation can be non-specific^2^ as in the case presented. Definitive diagnosis often requires advanced imaging such as computed tomography or MRI, or invasive testing such as biopsy or aspiration with culture, although ultrasound^12^ and plain radiography^13^ are potentially useful in the initial evaluation. As in the case presented, ultrasound has some role in identifying subperiosteal abscess.^14^

Documented patient informed consent and/or Institutional Review Board approval has been obtained and filed for publication of this case report.
